# Viability testing of material derived from *Mycobacterium tuberculosis *prior to removal from a Containment Level-III Laboratory as part of a Laboratory Risk Assessment Program

**DOI:** 10.1186/1471-2334-5-4

**Published:** 2005-01-24

**Authors:** Kym S Blackwood, Tamara V Burdz, Christine Y Turenne, Meenu K Sharma, Amin M Kabani, Joyce N Wolfe

**Affiliations:** 1 National Reference Centre for Mycobacteriology, National Microbiology Laboratory, Public Health Agency of Canada, Winnipeg, Manitoba, Canada

## Abstract

**Background:**

In the field of clinical mycobacteriology, *Mycobacterium tuberculosis *(MTB) can be a difficult organism to manipulate due to the restrictive environment of a containment level 3 (CL3) laboratory. Tests for rapid diagnostic work involving smears and molecular methods do not require CL3 practices after the organism has been rendered non-viable. While it has been assumed that after organism deactivation these techniques can be performed outside of a CL3, no conclusive study has consistently confirmed that the organisms are noninfectious after the theoretical 'deactivation' steps. Previous studies have shown that initial steps (such as heating /chemical fixation) may not consistently kill MTB organisms.

**Methods:**

An inclusive viability study (*n *= 226) was undertaken to determine at which point handling of culture extraction materials does not necessitate a CL3 environment. Four different laboratory protocols tested for viability included: standard DNA extractions for IS6110 fingerprinting, crude DNA preparations for PCR by boiling and mechanical lysis, protein extractions, and smear preparations. For each protocol, laboratory staff planted a proportion of the resulting material to Bactec 12B medium that was observed for growth for 8 weeks.

**Results:**

Of the 208 isolates initially tested, 21 samples grew within the 8-week period. Sixteen (7.7%) of these yielded positive results for MTB that included samples of: deactivated culture resuspensions exposed to 80°C for 20 minutes, smear preparations and protein extractions. Test procedures were consequently modified and tested again (*n *= 18), resulting in 0% viability.

**Conclusions:**

This study demonstrates that it cannot be assumed that conventional practices (i.e. smear preparation) or extraction techniques render the organism non-viable. All methodologies, new and existing, should be examined by individual laboratories to validate the safe removal of material derived from MTB to the outside of a CL3 laboratory. This process is vital to establish in house biosafety-validated practices with the aim of protecting laboratory workers conducting these procedures.

## Background

*Mycobacterium tuberculosis *(MTB), the causative organism of tuberculosis, has the distinction of repeatedly being ranked within the top five most commonly laboratory-acquired infections (LAIs) [1-3]. In 1976, Robert Pike prepared an extensive summary based on both published reports and surveys of 3921 LAIs that included both *M. tuberculosis *and other pathogens as the infectious agent [3]. He reported that laboratory and mortuary workers that are exposed to tubercle material have a TB incidence rate three times higher than that of the general population and indicated that only 18% of infections could be traced back to a known event. In 1987, a 25 year review at the National Animal Disease Center (NADC) described while only 35% of infections at the strict Biological Laboratory at Fort Detrick, MD, had a reportable, documented cause, the NADC could not account for 73% of LAIs occurring at its own facility [2]. With these reported statistics, it is negligent not to consider aerosol exposure in the absence of a known infecting episode, such as a needle prick [2]. A more recent report from 2003 demonstrated rates from 2 to 6.6 % of TB conversion among heath care workers (HCWs) in New York [4], in spite of current knowledge on precautions and safety measures in place. Furthermore, surveys suggest actual incidence of LAIs with MTB is greater than the amount of reported cases illustrate: these occurrences are likely underestimated due to the nature and length of the disease progression (i.e. workers move or retire before becoming symptomatic), and underreported to the social stigma attached [1,3,5].

Due to the nature of this organism, containment level three (CL3) laboratory operational and physical requirements have been recommended for manipulation of the live organism in North America [6]. Therefore, one would hypothesize that working in a CL3 with personal protective equipment including a respirator would be adequate to protect the worker. However, since conversions are still occurring, it is appropriate to consider the possibility that the procedure one is using to deactivate and extract material from the organism is not 100% efficient.

Currently, the application of molecular methodologies for rapid diagnostics of MTB, such as nucleic -acid amplification based identification and subtyping schemes, in addition to extensive genomic and proteomic research in this area, necessitates the removal of material derived from this organism out of a CL3 laboratory to perform the work in a less restrictive containment level 2 (CL2) rated area. Commonly, due to both limited CL3 space, costs and preventative maintenance needs, high-tech equipment such as liquid handling robots and sequencers are shared and can be housed in a central "DNA core" CL2 laboratory.

To consider the biosafety impact of removing organism material from a CL3 and manipulating it in a CL2, part of a risk assessment undertaken included the review of current literature on decontamination verification and viability testing of *M. tuberculosis*. The existing literature is limited in regards to viability testing of material derived from MTB with respect to safe manipulation outside of a Biological Safety Cabinet (BSC). To date, no conclusive study has confirmed that this organism is noninfectious after theoretical 'deactivation' steps. A few reports concerning survival after different heating kill treatments for DNA extractions [7-9], heat fixing of smears [10,11] and chemical fixation [12] were found.

Since, by definition, a risk assessment is based on, but not limited to, the properties of the agent used, personal risk factors, manipulation techniques, and the training and experience of staff, it is necessary to develop a method encompassing these factors to validate the safe removal of material derived from MTB from a CL3 laboratory for use in other laboratory areas. This approach was applied to all our methodologies and took into consideration variables such as interpersonal technique, culture load, temperature fluctuations and statistical significance.

## Methods

**Figure 1:**
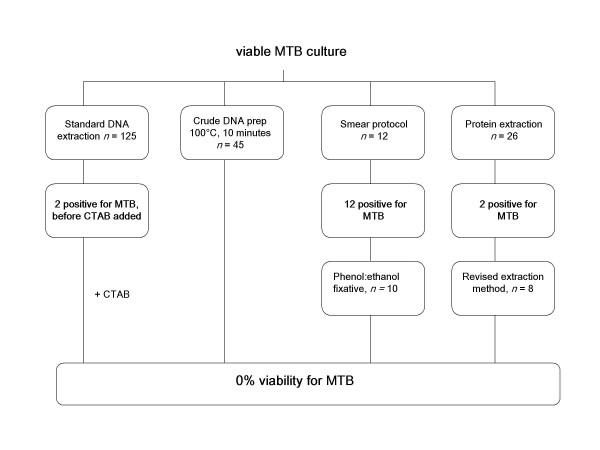
Overall study flow chart with results after eight weeks incubation of material at 37°C

The current viability study consisted of the evaluation of a total of 226 material extracts, consisting of 208 initial extracts and 18 extracts tested after revision of faulty protocols (figure 1). For each test performed, laboratory staff members sub-cultured a proportion (100 uL) of the resulting material to Bactec 12B radiometric medium (Becton Dickinson, Oakville, ON) that was kept for eight weeks to observe for growth. The vials were incubated at 37°C and the growth index (GI) was read weekly on BACTEC 460 machines. All vials with positive GIs were sub-cultured to tryptic soy agar (TSA) with 5% sheep blood, Middlebrook 7H10 agar and stained with the Kinyoun method for the presence of acid-fast bacilli (AFB). Those positive for AFB were examined for the presence of MTB using a DNA probe specific for *M. tuberculosis *complex, Accuprobe (GenProbe, San Diego, CA). Methods that resulted in viable MTB were revised and retested. All work completed was performed in a CL3 environment, using Class II Type B2 biosafety cabinets (BSCs), and personal protective equipment (PPE) including N100 particulate respirators, double gloves, and protective gowns. The following describe the protocols used in our laboratory at the start of this study.

### DNA extraction for IS6110 fingerprinting

This was performed according to the standard protocol [13]. A total of 125 Bactec 12Bs were inoculated with 0.1 mL of lysate materials. For initial heat deactivation steps, 1.5 mL screw-cap tubes were placed in a water-bath maintained at 80°C. The tubes were not submerged. Three technicians normally performing the procedure inoculated 12B media at different steps of the lysate protocol. Twenty-four samples were processed up to the point of lysozyme and proteinase K addition, prior to CTAB addition, and tested for viability. In 30 other samples the protocol was continued with the addition of choroform:isoamyl alcohol and subsequently centrifuged into organic and aqueous phases. For 10 samples, the organic (bottom) layer that is normally discarded was inoculated into 12B vials and the remaining 20 samples had their aqueous (top) layer planted. Sixty-one samples were processed to completion and inoculated into 12B vials. Finally, 10 extracts from frozen storage (-20°C) were retroactively tested by inoculation to 12B media.

### Crude lysate preparation

A total of 45 lysates used for PCR testing were tested for viability. Thirty-six lysates were prepared from actively growing cultures by three technicians that normally perform the testing procedure. Briefly, a loopful of culture is placed in a pyrex glass bottle containing 1 mL distilled water and glass beads and vortexed in a BSC. Generally this suspension has a turbidity of > 1 McFarland. This suspension is transferred to a screw-capped vial and is placed in a boiling water bath for 10 minutes followed by transfer into a tube containing 0.5 mm silica beads and mechanically lysed for 2 minutes with a Mini-8-Beadbeater (BioSpec Products, Bartlesville, OK). The resulting lysate is spun down for removal of debris and the supernatant transferred to a new tube to be used for PCR. Lysates were processed in duplicate and the position in water bath was noted (i.e. periphery or centre). A proportion of lysates (100 uL) were planted pre and post bead-beating. An additional nine previously frozen lysates stored at -20°C were also inoculated into 12B media (*n = *45 lysates).

### Smear preparation

Slides were prepared in duplicate according to our standard protocol: a loopful of organism is suspended in water with beads, vortexed and one drop added to a glass slide containing one drop of 0.5% phenol serum (made in-house). The slides were allowed to dry and were placed on a 95°C slide-warmer (Lab-Line Instruments, Melrose Park, IL) for 15, 30, 45 minutes, 1, 1.5 and 2 hours (*n = *12 slides). The heat fixed slide material was emulsified using a sterile swab and sterile distilled water. The suspension was transferred to a pyrex glass bottle containing 1 mL sterile water with beads, vortexed and 100 μl was inoculated into a 12B media vial.

### Protein extraction

Twenty-six extractions of *M. tuberculosis *organism were processed as follows. A volume of 25 mL of Middlebrook 7H9 broth was inoculated and incubated at 37°C for 20 days. The culture was centrifuged at 1900 × *g *for 15 minutes at room temperature and the pellet was resuspended with 2 ml of ice-cold phosphate buffer solution (PBS) pH 6.8 supplemented with 1% Tween-80. The sample was centrifuged as before at 4°C and the pellet washed twice in ice-cold 1% Tween-80 in PBS. Silicon beads (0.5 mm) and 500μl lysis buffer (2% CHAPS, 2% Triton X-100, 9.5 M urea and 1% DTT/TBP in water) were added to the pellet. The sample was mechanically lysed for 30 seconds and cooled on ice for 30 seconds, repeated eight times. The sample was then filter sterilized with a PES membrane, 0.22 μm Millipore filter unit (Millipore, Etobicoke, ON) into a 2 ml microcentrifuge tube on ice and stored at -80°C.

## Results

In total, 226 samples were tested for viability. As described below, 21 samples grew within the 8-week period, with 16 of those samples from three separate procedures yielding positive growth for *M. tuberculosis *(figure 1).

Of the 125 RFLP lysates tested, 2 of 24 sample materials that were tested for viability before addition of CTAB remained viable for *M. tuberculosis*. Following the complete extraction protocol as described [13], cultured samples yielded 0% viability for *M. tuberculosis*. Four of ten 12B media vials planted with lysates from frozen storage did show growth, and upon further investigation were determined to be contaminants (Table 1). All other vials had negative GI readings after 8 weeks of incubation.

**Table 1:** Results of positive Bactec 12B vials (*n *= 21).

**Description of material**	**Growth on TSA + 5% SBA @ 48 hrs**	**Growth on Middlebrook 7H11**	**Kinyoun Stain direct from positive 12B**	**Probe**	**Conclusion**
RFLP lysate – before CTAB addition (2 vials)	**-**	**+**, MTB morphology	AFB+ (4+), serpentine cording	ND	**Viable MTB^1^**
RFLP lysate from frozen storage #1	**-**	**+**, KS of growth AFB -	4 AFB/slide, morph not consistent with TB	MTB-	contaminant present – ubiquitous mycobacteria
RFLP lysate from frozen storage #2	**-**	**+**, KS of growth AFB -	15 AFB/slide, morph not consistent with TB	MTB-	contamination present – ubiquitous mycobacteria
RFLP lysate from frozen storage #3 & #4	**-**	**+**, KS of growth AFB -	No AFB		contamination present
Boiled lysate	**-**	**+**, Smooth colonies not consistent with MTB	AFB+ 5–10/field, clumping, no obvious serpentine cording	MTB-	ubiquitous mycobacteria
Smear, 1 h at 95°C & Smear, 2 h at 95°C^2^	**-**	**+**, MTB morphology	AFB+ (4+), serpentine cording	MTB+	**Viable MTB**
Protein extracts (2)	**-**	+, MTB morphology	AFB+ (4+), serpentine cording	MTB+	**Viable MTB**

^1 ^Accuprobe was not performed on these isolates, as MTB was expected due to morphology observed in addition to data from prior studies [7].

^2 ^All 12Bs from smears (*n *= 12) were positive, only 2 were chosen as representative for further testing Definitions: KS = Kinyoun stain, AFB = acid-fast bacilli, MTB = *M. tuberculosis*

Forty-five vials of TB PCR lysates were tested for viability. With the exception of one sample that was positive for growth and was later shown to be a contaminant (Table 1), all were negative for viable *M. tuberculosis*.

All vials containing suspensions from slide material that had been incubated for less than 1 hour on the slide-warmer exhibited identical growth rates, with a positive 12B vial in two weeks. Vials containing suspensions from slides that were incubated greater than 1 hour exhibited identical growth rates and had a positive 12B vials in 3–4 weeks. All 12 12B vials were positive for growth at 4 weeks incubation. Two representative vials were chosen for further analysis (1 hour and 2 hour slide incubations). These 12B culture vials were confirmed positive for *M. tuberculosis *complex using Accuprobe, in addition to displaying colony morphology consistent with TB on subculture to Middlebrook 7H10 agar and being AFB positive with the Kinyoun stain.

Two of 25 vials tested for viability from protein extractions became positive. Confirmation of viable MTB in these test vials was confirmed as described above. Based on these results, the methods for slide fixation and protein extraction were modified. Slides were fixed prior to staining using 5% phenol in ethanol according to Chedore *et al*. [10], and protein extractions samples were centrifuged at 4°C after lysis steps to rid the supernatant of cell debris before filter sterilization. These modified tests were planted again for growth of MTB, resulting in 0% organism viability for every test performed by each laboratory worker (Table 1). To date, ongoing viability testing of these tests have not shown any growth (data not shown).

## Discussion

There are few publications that review the efficacy of methodologies that render material extracts from a *M. tuberculosis *culture non-viable [7-10]. One such study by Bemer-Melchior *et al*. was prompted by a case of pulmonary tuberculosis acquired by a laboratory technician performing the standard method for IS6110-RFLP in a CL3 mycobacteriology [7]. In this study, placing the organisms in 80°C for 20 minutes gave breakthrough growth, which was also observed in our laboratory. Conflicting information exists which claims 100% loss of viability using this method, however, this report outlines potential reasons for the difference, such as the complete submersion of sample tubes, or volume and density of the suspension [9].

Bemer-Melchior *et al*. also found that submerging the culture at 100°C for 5 minutes rendered the sample completely non-infectious; data that was supported by work previously done in 1994 by Zwadyk *et al. *[8]. Although these studies were the first of its kind in verifying the safety of laboratory protocols and addressed issues regarding temperature, timing, cell density and sample volume in deactivating MTB cultures, they clearly demonstrated that certain methods of heating may not be efficient in complete sterilization of a MTB culture. With the unique cell wall characteristics and ability of MTB to clump (or cord), variables to be considered include cell mass density, actual temperature reached and actual time exposed to the heat source. Zwadyk *et al. *showed that using a 95°C heat block failed to completely inactivate the culture, and in fact, using an internal temperature probe demonstrated that the tube did not reach the intended temperature even after 20 minutes [8]. Another study issued a caution in removing MTB fixed with 0.5 – 1% glutaraldehyde from a CL3 as the process used to sterilize the culture failed [12]. Again, both the efficacy of rendering MTB material non-viable as well as the effect of the clumping or cording factors not being known was questioned. Therefore, testing all deactivation methods was recommended for all preparations in this laboratory [12]. In preparing a risk assessment for our CL3, it became evident that this was a necessary course of action to reduce the risk of LAIs for both CL3 and CL2 mycobacteriology staff as well as other non-mycobacteriology laboratory staff in the shared CL2 environment.

Genomic extractions, the most commonly performed test in our laboratory, are conducted using the standardized method outlined in 1997 by Van Embden *et al *for the purpose of RFLP typing [13]. Prior viability testing of this method in our lab showed that lysates contained viable MTB with the initial deactivation steps of this protocol, heating for 20 minutes at 80°C with addition of lysozyme and proteinase K, and thus could not be removed from containment until DNA extraction with CTAB. It is presumable that the small rate of viability observed (2 of 24), which is similar to what was seen by Bemer-Melchior *et al*., resulted from incompletely submerging sample tubes or cell density within the tube [9]. However, phenol-chloroform extraction such as the CTAB method and heating of specimens should be lethal to mycobacteria since phenolic-based disinfectants have been shown to be tuberculocidal [14], and to date our results reflect this fact.

To both preserve the integrity of genomic DNA for the use of fingerprinting and allow the sample to be further processed safely outside a CL3 laboratory, it is recommended to complete DNA extractions with CTAB as per the standard protocol to confirm the complete inactivation of *M. tuberculosis*. Further sampling of the DNA extractions performed in our laboratory (10% of each lysate batch) is ongoing for quality assurance purposes, i.e. to confirm integrity of reagents as well as to monitor staff performance and adherence to protocol. In addition, continuous sampling attaches statistical significance to the claim that this method ensures that the extracted material is 100% non-viable and guarantees staff safety.

For procedures that do not require intact, high quality DNA such as PCR testing, our laboratory depends on the much more expedient lysate method of boiling culture at 100°C for 10 minutes, followed by mechanical lysis for 2 minutes to release DNA. Although crude, this procedure is adequate for our PCR testing needs, has been shown to completely destroy live organism in our laboratory, and is consistent with other studies [7,8]. The study by Zwadyk *et al. *concluded that inactivating mycobacteria by heat lysing at a temperature of 100°C for 30 minutes did not inhibit its ability to be amplified by PCR or strand displacement amplification [8]. Furthermore, this study has shown that inoculating the boiled lysate alone without mechanical lysis was adequate in rendering the sample non-viable.

The viability testing of the two methods outlined above for DNA extractions were performed by various technicians who routinely follow these procedures, lending interpersonal variability to the study. It was demonstrated that the small nuances to procedure, such as the varying density of culture used, did not affect the method employed to deactivate the organism.

The last routine test assessed for organism viability was the slide preparation. While phenolics are known to be tuberculocidal, the use of phenol serum alone or in conjunction with heat in slide preparation is not sufficient for killing of the live organism. Flaming of the slide was not an option due to facility requirements that prohibit open flames inside a BSC, despite this, flaming of smear material was found in prior studies to unsuccessfully inactivate smear material [10]. The primary method for slide fixation was a 2-hour incubation at 95°C on the slide-warmer, which was assumed adequate for staining purposes. Slides were then transported to an area where respirator usage was not mandatory. It was discovered that every slide preparation was positive for viable TB. As a consequence of the viability testing and subsequent risk analysis, the protocol was altered to include chemical fixation with a 5% fixative of phenol:ethanol [10]. This allows lab staff to safely remove slides from a CL3 area and examine slides under a microscope without interference by the need to wear a bulky respirator.

Examining laboratory protocols for staff and building safety should be an integral part of any CL3 laboratory program. Implementation of a policy to test all procedures used routinely for viability in addition to new methodologies is highly recommended. As an example, applying this policy to a new procedure being utilized in the research arm of our laboratory proved valid: a protein extraction method being developed initially showed that of 2/26 tested samples yielded viable MTB. The researcher concluded that it was due to a clogging of the filter from large particles in the lysed culture used to isolate the proteins and revised the protocol to centrifuge the mixture after final lysis steps to remove cellular debris before filtration. This has proven successful so far, and continuous sampling of extracts is ongoing before removing the material from the CL3. To date, all have been negative.

## Conclusions

It is imperative to evaluate and record the actual rate of viability of DNA lysates from deactivated MTB cultures in individual laboratory settings [12]. This includes all material removed from the CL3 area. This process is vital to establish biosafety validated procedures and practices to protect laboratory workers conducting these procedures [2].

## Competing interests

The author(s) declare that they have no competing interests.

## Authors' contributions

KB carried out the studies involving genomic DNA extractions and drafted original and final manuscripts. TB tested all smear preparations, compiled viability data and assisted with the writing of the manuscript. MS contributed by testing protein extractions and writing the method used. CT contributed by testing of crude lysates and editing of manuscript. MS contributed by testing protein extractions and writing the method used. AK and JW conceived of the study, and participated in its design and ongoing coordination. All authors read and approved the final manuscript

## Pre-publication history

The pre-publication history for this paper can be accessed here:



## References

[B1] Collins CH, Kennedy DA (1998). Laboratory-acquired Infections: History, Incidence, Causes and Prevention.

[B2] Miller CD, Songer JR, Sullivan JF (1987). A twenty-five year review of laboratory-acquired human infections at the National Animal Disease Center. Am Ind Hyg Assoc J.

[B3] Pike RM (1976). Laboratory-associated infections: summary and analysis of 3921 cases. Health Lab Sci.

[B4] Garber E, San Gabriel P, Lambert L, Saiman L (2003). A survey of latent tuberculosis infection among laboratory healthcare workers in New York City. Infect Control Hosp Epidemiol.

[B5] Pike RM (1979). Laboratory-associated infections: incidence, fatalities, causes, and prevention. Annu Rev Microbiol.

[B6] U.S.Department of Health and Human Services Public Health Service Centres for Disease Control and Prevention and National Institutes of Health (1995). Primary Containment for Biohazards: Selection, Installation and Use of Biological Safety Cabinets.

[B7] Bemer MP, Drugeon HB (1999). Inactivation of Mycobacterium tuberculosis for DNA typing analysis. J Clin Microbiol.

[B8] Zwadyk P, Down JA, Myers N, Dey MS (1994). Rendering of mycobacteria safe for molecular diagnostic studies and development of a lysis method for strand displacement amplification and PCR. J Clin Microbiol.

[B9] Doig C, Seagar AL, Watt B, Forbes KJ (2002). The efficacy of the heat killing of Mycobacterium tuberculosis. J Clin Pathol.

[B10] Chedore P, Th'ng C, Nolan DH, Churchwell GM, Sieffert DE, Hale YM, Jamieson F (2002). Method for inactivating and fixing unstained smear preparations of mycobacterium tuberculosis for improved laboratory safety. J Clin Microbiol.

[B11] Kao AS, Ashford DA, McNeil MM, Warren NG, Good RC (1997). Descriptive profile of tuberculin skin testing programs and laboratory-acquired tuberculosis infections in public health laboratories. J Clin Microbiol.

[B12] Schwebach JR, Jacobs WR, Casadevall A (2001). Sterilization of Mycobacterium tuberculosis Erdman samples by antimicrobial fixation in a biosafety level 3 laboratory. J Clin Microbiol.

[B13] van Embden JD, Cave MD, Crawford JT, Dale JW, Eisenach KD, Gicquel B, Hermans P, Martin C, McAdam R, Shinnick TM, Small PM (1993). Strain identification of Mycobacterium tuberculosis by DNA fingerprinting: recommendations for a standardized methodology. J Clin Microbiol.

[B14] Rutala WA, Cole EC, Wannamaker NS, Weber DJ (1991). Inactivation of Mycobacterium tuberculosis and Mycobacterium bovis by 14 hospital disinfectants. Am J Med.

